# Influence of Patient Characteristics and Psychological Needs on Diabetes Mobile App Usability in Adults With Type 1 or Type 2 Diabetes: Crossover Randomized Trial

**DOI:** 10.2196/11462

**Published:** 2019-04-30

**Authors:** Helen NC Fu, Terrence J Adam, Joseph A Konstan, Julian A Wolfson, Thomas R Clancy, Jean F Wyman

**Affiliations:** 1 Center for Aging Science and Care Innovation School of Nursing University of Minnesota Minneapolis, MN United States; 2 Department of Pharmaceutical Care & Health Systems College of Pharmacy University of Minnesota MInneapolis, MN United States; 3 Institute of Health Informatics University of Minnesota Minneapolis, MN United States; 4 Department of Computer Science and Engineering University of Minnesota Minneapolis, MN United States; 5 School of Public Health University of Minnesota Minneapolis, MN United States; 6 Center for Nursing Informatics School of Nursing University of Minnesota Minneapolis, MN United States

**Keywords:** mHealth, diabetes, self-management, usability, Self-Determination Theory, mobile apps, user satisfaction

## Abstract

**Background:**

More than 1100 diabetes mobile apps are available, but app usage by patients is low. App usability may be influenced by patient factors such as age, sex, and psychological needs.

**Objective:**

Guided by Self-Determination Theory, the purposes of this study were to (1) assess the effect of patient characteristics on app usability, and (2) determine whether patient characteristics and psychological needs (competence, autonomy, and connectivity)—important for motivation in diabetes care—are associated with app usability.

**Methods:**

Using a crossover randomized design, 92 adults with type 1 or 2 diabetes tested two Android apps (mySugr and OnTrack) for seven tasks including data entry, blood glucose (BG) reporting, and data sharing. We used multivariable linear regression models to examine associations between patient characteristics, psychological needs, user satisfaction, and user performance (task time, success, and accuracy).

**Results:**

Participants had a mean age of 54 (range 19-74) years, and were predominantly white (62%, 57/92), female (59%, 54/92), with type 2 diabetes (70%, 64/92), and had education beyond high school (67%, 61/92). Participants rated an overall user satisfaction score of 62 (SD 18), which is considered marginally acceptable. The satisfaction mean score for each app was 55 (SD 18) for mySugr and 68 (SD 15) for OnTrack. The mean task completion time for all seven tasks was 7 minutes, with a mean task success of 82% and an accuracy rate of 68%. Higher user satisfaction was observed for patients with less education (*P*=.04) and those reporting more competence (*P*=.02), autonomy (*P*=.006), or connectivity with a health care provider (*P*=.03). User performance was associated with age, sex, education, diabetes duration, and autonomy. Older patients required more time (95% CI 1.1-3.2) and had less successful task completion (95% CI 3.5-14.3%). Men needed more time (*P*=.01) and more technical support than women (*P*=.04). High school education or less was associated with lower task success (*P*=.003). Diabetes duration of ≥10 years was associated with lower task accuracy (*P*=.02). Patients who desired greater autonomy and were interested in learning their patterns of BG and carbohydrates had greater task success (*P*=.049).

**Conclusions:**

Diabetes app usability was associated with psychological needs that are important for motivation. To enhance patient motivation to use diabetes apps for self-management, clinicians should address competence, autonomy, and connectivity by teaching BG pattern recognition and lifestyle planning, customizing BG targets, and reviewing home-monitored data via email. App usability could be improved for older male users and those with less education and greater diabetes duration by tailoring app training and providing ongoing technical support.

## Introduction

### Background

Patients with diabetes may benefit from self-management interventions to prevent complications including stroke and vision loss. Individuals with poor diabetes management have 2.3 times higher health care expenditures compared to those without diabetes [1]. Adhering to medical nutritional therapy and choosing what to eat are challenging for many patients with diabetes [2]. Using a diabetes app to record diet and track blood glucose (BG) shows promise to increase diet and medication adherence [3]. Small trials showed that using a diabetes app can improve glycemic control with a 0.4% to 1.9% reduction in hemoglobin A_1c_ (HbA_1c_) levels [4-6], but few patients use apps, possibly due to design problems affecting usability [7]. Diabetes app usability is the degree to which a user (patient) feels satisfied and finds the experience to be efficient and effective to accomplish tasks such as tracking BG readings [8].

Patient characteristics are hypothesized to influence user experience, but there is limited evidence on how demographic and clinical characteristics affect app usability [9-11]. Patients aged 56 years and older tend to report lower user satisfaction [12]. Prior studies also noted women made more errors than men when entering BG readings [10]. Technology experience and confidence also influenced patient ability to use diabetes apps [13]. For two popular apps, Glucose Buddy and MyFitnessPal, designs were not tailored based on patient knowledge and technology ability, which led to complaints of the apps being too complicated [13]. Most apps provide information input and output only and are limited in their theoretical basis [14]. In prior usability studies, the frameworks of health behavior theories were not considered in relation to understanding patient perspectives in app use. Assessing app usability and its relationship with patient characteristics and health behavior needs will fill critical knowledge gaps in user-centered design and best practices to promote optimal diabetes self-management. To fill this gap, we used a health behavior theory focused on motivation as the framework to understand the mechanism of how patient factors can influence the use of diabetes apps.

### Theoretical Framework

**Figure 1:**
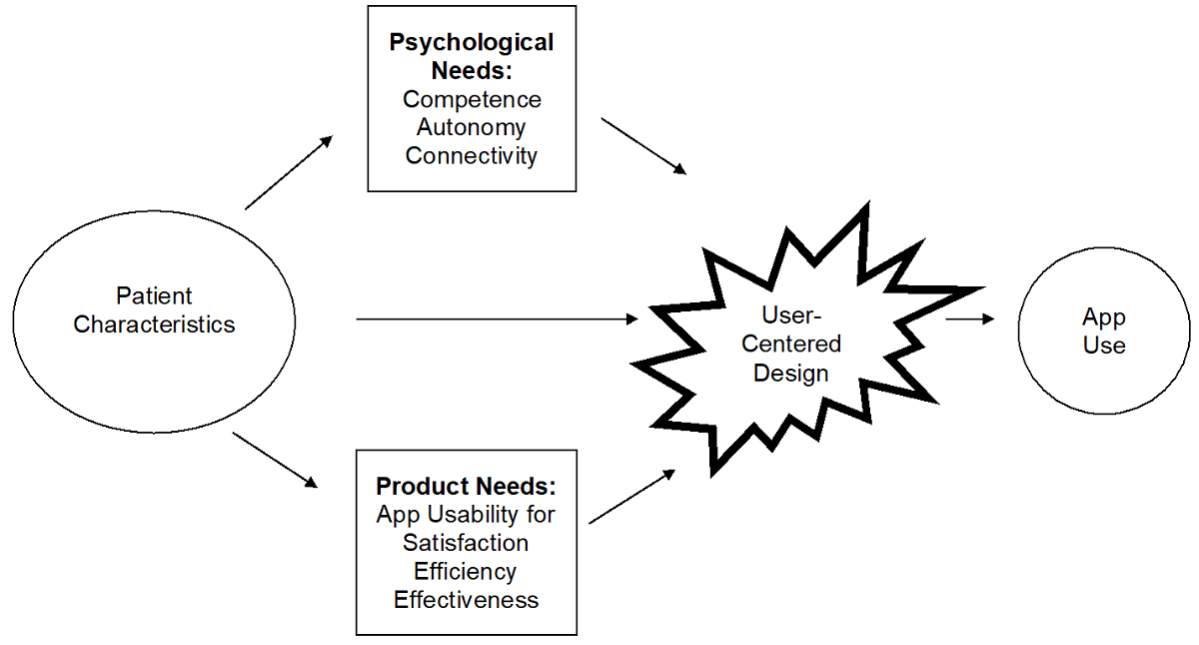
Usability model of diabetes app use.

We used Self-Determination Theory (SDT) on motivation to guide our hypotheses regarding how app functions and psychological needs influence app usability. Previous studies have shown that psychological needs are associated with adherence of healthy behaviors [15-18]. The motivation to adhere to healthy behavior is facilitated when patients experience satisfaction in three psychological needs: competence, autonomy, and relatedness [19]. Intrinsic motivation occurs when patients endorse personal benefits of healthy behaviors [20], which means that if patients perceive personal benefits of app use to assist them in adopting healthy behaviors, they can be motivated to use apps. Individual patient characteristics play a role in psychological needs and product needs that subsequently contribute to user-centered design and app use (Figure 1). Competence is the patient’s desire to be competent and experience confidence in keeping their BG in range [18]. App use can increase competence by displaying a report of out-of-range BG readings to increase patient understanding of BG numbers. Autonomy is a patient’s desire for empowerment in having options to change behaviors [20]. Using apps can increase autonomy by providing BG reports and carbohydrate (carb) intake patterns for all meals. Patients can visualize which meals require better carb control and health behavior changes in diet, insulin dose, or activity level. Relatedness or connectivity concerns the patient’s desire to be cared for by someone they trust [21]. Patients are more likely to adopt behaviors when they receive autonomous support and feel connected with people they trust such as a health care provider [20]. Apps can help patients connect with health care providers by supporting email communication and sharing home-monitored data.

### Objectives

The overall goal of this study was to evaluate the influence of patient factors on the usability of two publicly available diabetes apps. Thus, we planned for two sets of usability observations (app A and app B) from the same patient to adjust for app design effects, ensuring the changes in usability ratings were effects of the patient’s characteristics. Aim 1 was to determine the relationships between patient characteristics (eg, age, sex, education, technology use, diabetes history, and motivation) and app usability. We hypothesized that patient characteristics would predict user satisfaction and user performance in task time, success, and accuracy. Aim 2 was to determine the relationship between psychological needs and app usability. We hypothesized that user satisfaction would be associated with the psychological needs for competence, autonomy, and connectivity with a health care provider—theoretical constructs from SDT on motivation.

## Methods

### Study Design

A randomized crossover design was used to test two Android apps (OnTrack and mySugr) listed as “the Best Diabetes Apps 2016” by *Healthline* *.* This within-subject design (one group of patients to test two apps) allowed collection of two sets of usability observations and required a smaller sample size compared to a parallel group design. This design also adjusts for app design effects without worrying about between-subject differences in a two-group comparison. The Android platform was selected because it has the greatest number of users (52.7%) [22]. Because age was a potential confounder correlated with usability scores [23], and patients aged 56 years and older tend to give a lower satisfaction rating in apps [12], we created two age-based strata: adults age 56 years and older and adults younger than 56 years. A statistician randomly assigned the app testing order of AB or BA within each age stratum using a computer software program and provided group assignment in a sealed opaque envelope. These steps were to adjust for app effects and address potential bias due to learning effects and investigator preferences. The primary usability outcome was user satisfaction measured by the System Usability Scale (SUS) [24]. Secondary usability outcomes were user performance, including efficiency (task time) and effectiveness (task success and accuracy). The University of Minnesota Institutional Review Board approved the study. All participants signed an informed consent document and received a US $50 honorarium.

### Participants

Participants were recruited by flyers posted at community or Veterans Affairs (VA) clinics, university campuses, community bulletin boards, diabetes support group meeting sites, and websites (Craigslist and Facebook). A total of 92 participants met all inclusion criteria: (1) age 18 years or older, (2) type 1 or type 2 diabetes, (3) insulin therapy for at least 6 months, (4) use of an Android mobile phone for at least 6 months to ensure familiarity with the testing app’s operating system, (5) English proficiency, (6) adequate vision to read email or text messages on their current mobile phone, and (7) mobile phone use proficiency. Individuals who used any diabetes app in the past 6 months or had ever used OnTrack or mySugr were excluded. Because OnTrack and mySugr are made available to the public, including patients with either type 1 or type 2 diabetes was important for generalizability. We verified mobile phone use proficiency by screening for app use and did not include typical mobile phone usage for calls, texting, emailing, or taking pictures.

### Procedures

In-person app training and testing were conducted from July 26 to November 30, 2017. Individual study sessions were held in a private room. The sessions ranged from 1 to 3.5 hours. During app training, participants watched YouTube tutorial videos posted by the app developers and practiced following a checklist for seven tasks on a Samsung 5S study phone: (1) enter a carb intake, (2) enter an exercise activity, (3) enter an insulin dose, (4) enter a BG reading, (5) locate a BG report for days of the week, (6) locate a BG report for each meal, and (7) email a BG report. During app testing, participants followed a checklist to test app tasks in a randomized order with data units that were different from app training. Each participant completed a SUS questionnaire at the end of each app test. During a 30-minute break between the first and second app test, participants completed a background survey and were given an opportunity to eat a light snack and use the restroom. App training and testing were conducted by the first author (HNCF), who kept field notes that included nonverbal reactions to app testing and open responses of app preference. Another researcher checked fidelity from the audio recording of the study sessions.

### Measurements

User satisfaction in app usability (primary outcome) was rated by the SUS, a 10-item questionnaire, at the end of app testing [24]. The SUS measures ease of use, app function integration, user confidence, learning needs prior to use, and app use intention. Survey items included “I felt very confident using this app” and “I would like to use this app frequently.” Responses are on a 5-point Likert scale (0=strongly disagree to 4=strongly agree). Even-numbered items are negative statements, such as “this app is unnecessarily complex.” The SUS is widely used in product usability evaluation with a reliability coefficient alpha of .91 [23] and a loading factor greater than 0.3 for construct validity [25]. Scores of 85 or higher are excellent; scores less than 70 are acceptable. Scores between 50 and 69 are marginally acceptable; scores 50 and lower are unacceptable [26]. Secondary outcomes were user performance in terms of efficiency (measured by task time) and effectiveness (measured by task success and accuracy). Task time is the total task completion time per app. Task success is the degree to which a user independently completed required tasks [27]. Each app task success was rated from 0% to 100%. The rating was zero when the app lacked a testing function or when a participant received more than 50% of standard technical support. The user success rate was calculated by averaging the success of all tasks. The user accuracy was whether the participant performed tasks correctly (eg, correct insulin dose) and was calculated by averaging the accuracy of all tested tasks.

Patient characteristics were self-reported in a 32-item background survey that included demographics (age, sex, race/ethnicity, and education), mobile phone brand, technology use, and diabetes factors (types, HbA_1c_, duration, insulin use, BG testing, and prescribed BG testing). An established motivation scale, the Treatment Self-Regulation Questionnaire (TSRQ), was also included, which assessed patients’ reasons for engaging in diabetes self-management behaviors with 8 items for intrinsic motivation and 11 items for extrinsic motivation rated on a 7-point Likert scale [18]. Both types of motivation scores were calculated by averaging the response ratings, which ranged from 1 (not true at all) to 7 (very true). Overall motivation was assessed by the Relative Autonomy Index, which was calculated by subtracting the intrinsic motivation score with the extrinsic motivation score. Positive scores indicate greater intrinsic motivation, whereas negative scores indicate greater extrinsic motivation. The TSRQ has been validated across settings and for other health behaviors with an internal consistency alpha coefficient greater than .73 in a prior study [28] and .82 in this study.

Competence in diabetes self-management was measured by the Perceived Competence Scale, which ranged from 1 (not true at all) to 7 (very true) on a 7-point Likert scale with four items and scored by averaging the responses [29]. Its internal consistency alpha coefficient was greater than .80 in a prior study [18] and .88 in this study. Autonomy in diabetes care was measured by four items designed by investigators and validated by four experts (endocrinologist, physician-researcher in diabetes, and two PhD-prepared diabetes nurse educators). This scale measured patient interest in identifying personal BG readings and carb intake trends, which is a subcomponent of autonomy [30]. Patients who desire autonomy (intrinsic motivation) are proactive in their health behavior and choices for diabetes management [30]. Responses were rated on a 5-point Likert scale rating from 1 (strongly disagree) to 5 (strongly agree); an overall score was obtained by averaging all item responses. The alpha coefficient of .74 was deemed acceptable. Health care provider connectivity was rated by the Health Care Climate Questionnaire, which assessed the degree to which primary health care providers offer autonomous support in diabetes management [31]. The score is based on the average response to six items on a 7-point Likert scale rating from 1 (strongly disagree) to 7 (strongly agree). Its reliability alpha coefficient was .82 in a prior study [31] and .94 in this study.

### Statistical Analysis

We targeted a sample size (n=92) for a regression model (n=84) with 10% attrition (n=8) based on 13 predictors with *R*^2^ correlation of .20 and alpha of .05 in the calculation. Residual plots showed no evidence of heteroskedasticity. Analyses of *t* tests and chi-square tests were used to assess differences between the two age strata and sex groups. Paired *t* tests of mySugr and OnTrack usability scores showed significant differences (*P*<.05), hence all regression analyses were adjusted for app group and testing order with an interaction term. We set an alpha of .05 for statistical significance. All analyses were performed using R statistical software [32].

For aim 1, both random effect (repeated app testing) and fixed effect (app group) were analyzed by a linear mixed effect multiple regression model of analysis of variance (ANOVA). The full model was run separately for each usability outcome model, including 15 predictors of patient characteristics. Mobile phone brand and education variables were collapsed into dichotomous variables: (1) Samsung versus not Samsung Android, and (2) high school education or less versus education higher than high school. For aim 2, the model for aim 1 was used by adding a psychological need predictor (eg, competence) while adjusting for covariates of patient characteristics (key demographics, technology factors, diabetes history, and motivation), testing order, app group, and an interaction term between testing order and app group. We also assessed the individual mediation effect of task time, success, and accuracy on user satisfaction to explain all or part of the relationship between the psychological need and user satisfaction [33].

## Results

### Sample Recruitment and Characteristics

Diverse recruitment sites yielded 92 participants who completed the study from urban and suburban Minnesota: 46 were recruited from Facebook (50%), eight from patient referrals (9%), seven from a community clinic (8%), six from a university (6.5%), six from public housing (6.5%), five from Craigslist (5%), four from a VA clinic (4%), three from diabetes support groups (3%), and seven from miscellaneous sites (8%). Participant characteristics are summarized in Table 1.

**Table 1:** Sample characteristics and psychological needs (N=92).

Characteristics/psychological needs		Participants
Age (years), mean (SD)		54 (13)
Men, n (%)		38 (41)
**Race, n (%)**		
	White	57 (62)
	Black/African American	23 (25)
	Native American	10 (11)
	Asians	2 (2)
**Highest completed education, n (%)**		
	Elementary	4 (4)
	High school or equivalent	27 (29)
	Community/technical school	31 (34)
	Bachelor’s degree	19 (21)
	Graduate degree	11 (12)
**Device brand, n (%)**		
	Samsung	44 (48)
	LG	19 (20)
	iPhone	8 (9)
	ZTE	7 (8)
	Motorola	6 (6)
	Other	8 (9)
**Mobile phone comfort level, n (%)**		
	Very uncomfortable	23 (25)
	Neither	12 (13)
	Comfortable	33 (36)
	Very comfortable	24 (26)
**Diabetes types, n (%)**		
	Type 1	28 (30)
	Type 2	64 (70)
HbA_1c_^a^ % (ranges 5-14), mean (SD)		8.2 (1.5)
Diabetes duration (years), mean (SD)		17 (11)
Insulin duration (years), mean (SD)		12 (12)
**Insulin use types, n (%)**		
	Insulin pump	14 (15)
	Long- and short-acting injection	46 (50)
	Long-acting injection	28 (30)
	Short-acting injection	2 (2)
	None (stopped use)	2 (2)
Blood glucose testing prescribed per day, mean (SD)		3.8 (1.8)
**Blood glucose testing per day, mean (SD)**		6.2 (1.4)
	Daily or less, n (%)	19 (21)
	2 times a day, n (%)	34 (37)
	4 times a day, n (%)	21 (23)
	>4 times a day, n (%)	18 (19)
Overall motivation^b^, mean (SD)		2.16 (1.3)
Intrinsic motivation, mean (SD)		5.43 (0.9)
Extrinsic motivation, mean (SD)		3.26 (1.2)
Competence, mean (SD)		5.38 (1.1)
Autonomy, mean (SD)		3.92 (0.6)
Connectivity with health care provider, mean (SD)		6.05 (1.2)

^a^HbA_1c_: hemoglobin A_1c_.

^b^Also known as the self-determination index obtained from intrinsic motivation score minus extrinsic motivation score.

More than half of the participants were women, nearly half used Samsung phones, and 70% (64/92) had type 2 diabetes. The mean age was 54 years (range 19-79) with a median age of 57, and the mean HbA_1c_ was 8.2% (range 5%-14%) or 66 mmol/mol (range 31-130). The only missing data was an HbA_1c_ level from one participant.

### App Usability

The overall mean user satisfaction was 62 (SD 18) and mean task completion was 7 (SD 3.8) minutes. Participants had a mean task success rate of 82% (SD 19%) and mean accuracy rate of 68% (SD 21%). Participants rated the two apps as marginally acceptable in user satisfaction (SUS scores between 50 and 69) as shown in Table 2. OnTrack scored 68, which is considered a “D” grade (eg, scores between 60 and 69); mySugr received a score of 55, which is an “F” grade (eg, scores less than 60). User performance was better for OnTrack compared to mySugr: more efficient (mean time 6.6, SD 3.7 minutes versus mean time 7.5, SD 3.7 minutes, *P*<.001), more effective (mean task success 84%, SD 18% versus 80%, SD 20%, *P*=.03), and more accurate (mean accuracy 74%, SD 20% versus 63%, SD 22%, *P*<.001).

### Patient Characteristics

Demographics, technology use, diabetes factors, and motivation were not predictors of user satisfaction as assessed by the SUS for the tested apps (Table 3). Age, sex, and education were predictors of user performance in task time and success rate. Adults older than 56 years took an extra 2.2 minutes (95% confidence interval [CI] 1.1-3.2) for task time, had lower task success rate (95% CI 3.5%-14.3%), and higher task error rate (95% CI 4.2%-16.4%) compared to adults aged 18 to 55 years. On average, for every 10 years of age, adult patients spent 0.8 minutes longer to use the app (*P*=.02), and the task success rate decreased by 4.6% (*P*=.003). Men were less proficient, took an extra 1.7 minutes (*P*=.01), and achieved 6.9% less success (*P*=.04) compared to women. Participants with education beyond high school had 6.4% less user satisfaction (*P*=.04) and greater success by 10.5% compared to participants who were not educated beyond high school (*P*=.003). Current Samsung mobile phone users were 7.3% more accurate (*P*=.05).

Diabetes type was not a predictor of task time or success, but diabetes duration negatively influenced user accuracy. The longer duration of diabetes, the less accurate participants were in using diabetes apps. A 10-year increase in diabetes duration was associated with an 8.5% drop in task accuracy (*P*=.02). A 10-year use of insulin increased accuracy by 7.1%, but it was not statistically significant (*P*=.06). Glycemic control of HbA_1c_ level showed no association with user satisfaction and performance. Self-reported BG testing frequency, prescribed BG testing frequency, and motivation for diabetes care were not associated with app usability.

**Table 2:** Diabetes app usability outcomes.

Usability	Overall (N=184), mean (SD)	mySugr (n=92), mean (SD)	OnTrack (n=92), mean (SD)	Difference (95% CI)	*P* value^a^
Practice time (minutes)	19 (8)	22 (9)	16 (6)	5.6 (4.0-7.2)	<.001
Satisfaction	62 (18)	55 (18)	68 (15)	12.7 (8.2-17.2)	<.001
Efficiency (minutes)	7.0 (3.8)	7.5 (3.8)	6.6 (3.7)	0.8 (0.3-1.3)	<.001
Success (%)	82 (19)	80 (20)	84 (18)	3.9 (0.3-7.5)	.03
Accuracy (%)	68 (21)	63 (22)	74 (20)	11.0 (6.0-16)	<.001

^a^Obtained from paired *t* test comparing two apps, mySugr and OnTrack*.*

**Table 3:** Adjusted associations between patient characteristics and app usability.

Predictors effect (coefficients)		Satisfaction (SUS)	Efficiency (minutes)	Success (%)	Accuracy (%)
**Characteristics**		Model 1	Model 2	Model 3	Model 4
	Age per 10 years	−0.5	0.8^a^	−4.6^b^	−2.5
	Men vs women	0.1	1.7^a^	−6.9^a^	−0.1
	>High school vs ≤high school^c^	−6.4^a^	−1.2	10.5^b^	0.3
	Samsung vs not Samsung	1.5	−0.8	5.3	7.3
	Mobile phone comfort	0.6	−0.1	0.6	−0.3
	Diabetes type 2 vs type 1	−5.5	1.6	−4.7	−7.4
	Diabetes duration per 10-year diagnosis	3.6	0.5	−0.1	−8.5^a^
	Insulin duration per 10-year use	−1.3	0.6	−3.1	7.1
	HbA_1c_^d^	0.4	−0.2	1.8	0.7
	Blood glucose testing per day	−0.4	0.2	−1.7	−1.0
	Blood glucose testing prescribed per day	−0.2	−0.2	0.9	−1.2
	Motivation (TRSQ^e^)	−0.4	−0.03	0.7	−0.1
Testing order		−3.9	−1.2	11.2^b^	3.6
App group		8.4^a^	−1.3^b^	9.8^b^	11.1^a^
Interaction order and app		8.3	0.9	−11.3	−0.5
Adjusted^f^ *R*^2^		.14	.35	.31	.17

^a^*P*<.05 statistical significance.

^b^*P*<.01 statistical significance.

^c^Highest completed education was high school or less.

^d^HbA_1c_: hemoglobin A_1c_.

^e^TRSQ: Treatment Self-Regulation Questionnaire.

^f^Obtained from linear regression model analysis without repeated measures.

### Psychological Needs

Psychological needs were significantly associated with user satisfaction but not associated with user performance. This supports our hypothesis that patient ratings of competence, autonomy, and health care provider connectivity are related to user satisfaction with diabetes apps. Patients who rated high in diabetes care competence were more satisfied with diabetes apps: a 1-unit increase in diabetes competence score was associated with an increase of the SUS score by 3.1 points (*P*=.02; Table 4). Similarly, patients who reported greater autonomy or interest to learn their personal BG and carb patterns were more satisfied with the apps: a 1-unit increase in the autonomy score was associated with an increase of the SUS score by 5.9 points (*P*=.006). Patients who rated a higher connectivity with health care providers (receiving greater autonomous support) expressed higher user satisfaction: a 1-unit increase of connectivity score was associated with an increased SUS score of 2.5 points (*P*=.03). Patient autonomy, as an interest in learning personal patterns of BG and carbs, was also associated with greater successful task completion by 4.9% (*P*=.049). The effect of psychological needs on user satisfaction was not strongly mediated by task time, success, and accuracy (percent mediated 0.5%-19.7%).

**Table 4:** Adjusted associations between psychological needs and app usability.^a^

Psychological needs (coefficients)		Satisfaction (SUS^b^)	Efficiency (minutes)	Success (%)	Accuracy (%)
**Competence**		Model 1A	Model 2A	Model 3A	Model 4A
	Adjusted effect	3.1^c^	0.2	−0.1	−2.9
	Adjusted^d^ *R*^2^	.16	.35	.31	.18
**Autonomy**		Model 1B	Model 2B	Model 3B	Model 4B
	Adjusted effect	5.9^e^	−0.8	4.9^c^	1.2
	Adjusted^d^ *R*^2^	.17	.37	.33	.17
**Connectivity**		Model 1C	Model 2C	Model 3C	Model 4C
	Adjusted effect	2.5^c^	0.2	−0.02	−0.01
	Adjusted^d^ *R*^2^	.16	.35	.31	.17

^a^N=184 observations from randomized 92 patients, adjusted all models with 15 covariates listed in model 1 from Table 3, which included age, sex, education, use of Samsung, mobile phone comfort, diabetes types, diabetes duration, insulin duration, hemoglobin A_1c_, blood glucose testing per day, blood glucose testing prescribed per day, motivation, testing order, app group, and interaction term between order and app.

^b^SUS: System Usability Scale.

^c^*P*<.05.

^d^Obtained from linear regression model analysis without repeated measures.

^e^*P*<.01.

## Discussion

### Psychological Needs and User Satisfaction

To our knowledge, this is the first study to report a relationship between app usability and the characteristics and psychological needs of the patient. A strength of our approach was the relatively large and diverse study population (N=92) for usability testing because most mHealth usability evaluations have fewer than 30 participants and limited recruitment sites. Our study population resided in both urban and suburban settings and included African Americans, Native Americans, and Asians. Study findings indicate that psychological needs and education are important factors in app usability, whereas patient characteristics are important for user performance or the ability to use an app efficiently, successfully, and accurately. Diabetes app usability, as assessed by user satisfaction (SUS), was associated with three psychological needs important for motivation in diabetes care: competence, autonomy, and connectivity with a health care provider.

Competence in diabetes care was associated with greater app user satisfaction. In our study, patients wanted to use the app to increase their competence and preferred the convenience to track data on the go. Apps offering out-of-range BG reports can help patients identify whether their meals, insulin dose, and physical activities need to be adjusted. This perceived app benefit agrees with prior research that patients desired educational information and goal setting in apps to help them plan self-management activities [13]. Autonomy in diabetes care, as assessed by patient interest in personal patterns, correlated with greater user satisfaction and successful task completion. This is consistent with prior research showing that a diabetes app can help patients set realistic goals based on personal patterns and see choices to modify behavior [13]. Patients wanted a customized care plan within an app to help them control diabetes and learn to improve eating habits [34]. Addressing patient desire or need to connect with a health care provider is important for patient engagement in an mHealth intervention. Connectivity with a health care provider was positively associated with user satisfaction. Patients who were well-connected and reported autonomous support from their health care provider rated higher satisfaction. This is consistent with other studies that found patients were more motivated and would use mHealth tools for diabetes when they perceived their health care providers to be autonomously supportive [15]. Apps facilitate data sharing and patient-provider communication. Clinicians can view analysis reports emailed to them or view them on patients’ mobile phones during clinic visits; having real-time data facilitates discussions with patients and pinpoints exact areas for behavior changes.

### Patient Characteristics and User Performance

Patient characteristics correlated with the individual’s ability to use an app. User performance in task time, success, and accuracy varied by age, sex, education, or diabetes duration when controlled for covariates (eg, education, diabetes types, HbA_1c_, BG testing, and motivation). A 10-year age increment was associated with a slower time performance of 0.8 minutes and lower success performance by 4.6%; surprisingly, age did not correlate with accuracy even though younger users are typically more accurate with technology use. This may be explained by the design of this study, which provided as much technical support and time as desired.

In contrast to prior studies, women outperformed men in time efficiency and task success when accounting for other participant factors. This sex difference may be related to differences in mobile phone and app use. According to one study that tracked 75,000 people’s use of popular websites and apps, women spend more time than men on mobile phones (49% versus 39%) [35]. Women also use social media apps (eg, Facebook) more often than men (83% versus 75%) [36]. We ran a separate full model adjusted for Facebook recruitment, which did not affect results. Education beyond high school was significantly correlated only with user success performance. This suggests that if participants with high school education or less are provided with technical support, they can learn to use an app as efficiently and accurately as those with more education.

Diabetes duration was significantly related to user accuracy. A 10-year diabetes history decreased accuracy by 8.5%, perhaps because of diabetes complications. The rate of diabetic peripheral neuropathy increases by twofold for those with diabetes for longer than 10 years [37]. The prevalence of diabetic retinopathy 10 years after diagnosis is 60% [38]. Finger nerve pain can make it hard and painful to tap correct app icons. Icons and fonts on a small mobile phone screen could be hard to read for those with vision complications from retinopathy. In this study, most participants had suboptimally controlled diabetes with an elevated mean HbA_1c_ level of 8.2% (66 mmol/mol). The target HbA_1c_ level for adults older than 65 years is less than 7.5% (58 mmol/mol) [39] and 7% or less (53 mmol/mol) for adults younger than 65 years without a history of hypoglycemia [2]. HbA_1c_ level, BG monitoring frequency, and motivation in diabetes care did not correlate with app usability.

### Clinical Implications

Our study provides new insights into the theoretical basis of health behavior in diabetes app usability. Application of SDT provided important insights on how patient needs and app designs are related. Psychological needs of competence, autonomy, and connectivity with a health care provider (motivational constructs) were associated with user satisfaction. These results suggest that clinicians should address these psychological needs when recommending the use of a particular diabetes app. Clinicians could help improve patient competence by providing education on BG and carb pattern recognition and planning for lifestyle modifications (eg, lowering carb intake). Clinicians who customize a care plan and a BG target range could help increase patient autonomy so that patients can set up a parameter in their apps to analyze BG accordingly. Autonomous support and home-monitored data received through email could promote connectivity with health care providers. Addressing psychological needs for competence, autonomy, and connectivity can potentially lead to long-term app use. Clinicians should screen for diabetes complications that may affect user accuracy.

### Limitations and Future Directions

Several limitations in this study provide directions for future research. We were only able to evaluate two diabetes apps in a single study session with findings applicable for a short-term app experience. User satisfaction may change with long-term app use. Future research should include long-term follow-up, record app adherence rate, and assess factors affecting whether or not long-term app use will be sustained.

Fatigue with the 2-hour testing session could have affected participant performance. However, we provided a 30-minute rest break that included refreshments, and none of the participants complained about being tired at study completion. Another possible study limitation was related to the sample population. We recruited a diverse sample with different proportions of nonwhite participants from sites such as public housing and a federally qualified health center. This heterogeneity in race breakdown by recruitment site made it challenging to distinguish between the effects of race and recruitment; thus, we were unable to include race/ethnicity as a covariate.

Unmeasured covariates, such as socioeconomic status (eg, income), types of medical insurance, diabetes complications, and obesity, could influence results. However, our study included multiple recruitment sites and a variety of patient backgrounds with different education levels, insulin use types (pump users on private insurance and those on injection therapy on a public insurance program), and different housing facilities. Covariates, such as education and mobile phone model, may count as a proxy for socioeconomic status. A multiple variable model accounted for common demographics and diabetes history. Our study excluded adolescents with diabetes as well as family caregivers. Future studies should recruit minority patients, adolescents, and caregivers. We did not include laboratory-based usability measures. Future studies can further identify app use barriers through other methods of quantifying usability problems (eg, recording screen reaction, counting keystrokes, and tracking eye movements).

### Conclusions

Applying SDT to diabetes app usability revealed that addressing psychological needs for diabetes care competence, autonomy, and connectivity with a health care provider may enhance patient motivation to use diabetes apps. Patient-centered training and ongoing technical support could improve usability for (1) older male users, (2) those with education levels of high school or less, and (3) those with a long duration of diabetes. User-centered apps are desired by patients. App designs and features should incorporate health behavior theoretical framework and be tailored to patients’ ages and abilities.

## Abbreviations

ANOVA: analysis of variance
BG: blood glucose
HbA_1c_: hemoglobin A_1c_
SDT: Self-Determination Theory
SUS: System Usability Scale
TSRQ: Treatment Self-Regulation Questionnaire
VA: Veterans Affairs

## Appendix

Multimedia Appendix 1 CONSORT‐EHEALTH checklist (V 1.6.1).
